# Supplementary material for: MetaRibo-Seq measures translation in microbiomes
Source: Nat Commun. 2020 Jun 29;11:3268. doi: 10.1038/s41467-020-17081-z (PMC7324362; doi:10.1038/s41467-020-17081-z)
Supplement: Supplementary file 10 — Supplementary Data 7 [file 41467_2020_17081_MOESM10_ESM.zip › File2/Confidence_High_Taxonomy/57001_out.krona.html]

Javascript must be enabled to view this page.

members
magnitude
magnitudeUnassigned
count
unassigned
taxon
rank

57001\_out

72

superkingdom
2
72

phylum
72
976

class
200643
72

order
72
171549

family
72
815

genus
816
72

species
67

SRS011084\_contig\_number\_2887SRS012902\_contig\_number\_303SRS013158\_contig\_number\_contig-100\_1681.1681SRS013215\_contig\_number\_69SRS014736\_contig\_number\_4150SRS015190\_contig\_number\_3006SRS015578\_contig\_number\_19077SRS015960\_contig\_number\_13722SRS016095\_contig\_number\_19030SRS016132\_contig\_number\_366SRS016267\_contig\_number\_1901SRS017622\_contig\_number\_299SRS018313\_contig\_number\_6275SRS018541\_contig\_number\_9015SRS018656\_contig\_number\_contig-100\_112.76535SRS018817\_contig\_number\_13166SRS018888\_contig\_number\_7003SRS018984\_contig\_number\_5542SRS019267\_contig\_number\_1064SRS019601\_contig\_number\_14450SRS019787\_contig\_number\_257SRS021484\_contig\_number\_4419SRS023346\_contig\_number\_1550SRS023829\_contig\_number\_41SRS024075\_contig\_number\_365SRS024388\_contig\_number\_5466SRS024549\_contig\_number\_530SRS043411\_contig\_number\_12080SRS043701\_contig\_number\_2973SRS046502\_contig\_number\_9238SRS046712\_contig\_number\_239SRS048981\_contig\_number\_2246SRS050941\_contig\_number\_17345SRS051610\_contig\_number\_3632SRS053573\_contig\_number\_1347SRS055966\_contig\_number\_38SRS055966\_contig\_number\_812SRS056259\_contig\_number\_12273SRS057717\_contig\_number\_1123SRS063127\_contig\_number\_2225SRS063190\_contig\_number\_3999SRS063985\_contig\_number\_21006SRS064276\_contig\_number\_27563SRS075398\_contig\_number\_1382SRS075773\_contig\_number\_7345SRS076804\_contig\_number\_7081SRS077194\_contig\_number\_13093SRS077589\_contig\_number\_contig-100\_111.43403SRS098644\_contig\_number\_31204SRS098655\_contig\_number\_3704SRS098881\_contig\_number\_15263SRS104036\_contig\_number\_3206SRS104485\_contig\_number\_682SRS104636\_contig\_number\_14893SRS142599\_contig\_number\_30132SRS143342\_contig\_number\_1463SRS143417\_contig\_number\_18130SRS144135\_contig\_number\_32519SRS144362\_contig\_number\_13598SRS146812\_contig\_number\_43186SRS147022\_contig\_number\_450SRS147272\_contig\_number\_356SRS148159\_contig\_number\_33232SRS149075\_contig\_number\_10346SRS893279\_contig\_number\_1290SRS893342\_contig\_number\_552SRS971275\_contig\_number\_11470
817

46506

SRS017521\_contig\_number\_19519SRS019397\_contig\_number\_556SRS143417\_contig\_number\_1221
3
species

species
1

SRS058723\_contig\_number\_1158
2291999

457392

SRS147557\_contig\_number\_28827
1
species
